# Prevention of Umbilical Sagging After Medium Definition Liposuction

**DOI:** 10.1093/asj/sjaa051

**Published:** 2020-02-17

**Authors:** Giuliano Borille, Patrícia M A Neves, Gustavo P Filho, Roy Kim, Gabriele Miotto

**Affiliations:** Division of Plastic and Reconstructive Surgery, Emory University School of Medicine, Atlanta, GA

## Abstract

**Background:**

The loss of the umbilical vertical axis, causing a horizontal shape deformity after liposuction, is a current aesthetic issue. The use of energy devices, such as LASER and VASER, has been advocated as an option for improving skin retraction, but no data are available on the prevention of umbilical sagging.

**Objectives:**

The authors sought to describe a technique for preventing umbilical deformities after medium definition liposuction employing suction-assisted liposuction.

**Methods:**

Over a period of 31 months, 62 patients underwent medium definition liposuction with direct needle fixation of the umbilical stalk to prevent horizontal umbilical deformities. All patients underwent surgery performed by a single surgeon (G.B.). All patients underwent objective measurements of the umbilical shape before and after the procedure utilizing digital image measurements by Mirror Image software, version 6.0 (Fairfield, NJ). Statistical analysis was performed with IBM SPSS Statistics V26. The mean age of the patients was 28.8 years. The follow-up evaluation was performed 2 weeks and 9 months postoperatively.

**Results:**

Over a period of 31 months, 60 patients (96.7%) who underwent abdominal etching liposuction showed maintenance of (n = 9, 14.5%) or improvements in the umbilical shape 9 months postoperatively (n = 51, 82.2%, *P* < 0.05). Two patients (3.2%) experienced worsening of the umbilical shape after surgery despite suture fixation.

**Conclusions:**

Horizontal shape deformities of the umbilicus after liposuction can be improved by utilizing direct needle fixation of the umbilical stalk. The approach has been shown to be effective, safe, and reproducible for the prevention of umbilical sagging in selected patients.

**Level of Evidence: 4:**

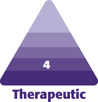

Since its inception in the 1980s, liposuction has undergone a tremendous paradigm shift from the simple removal of excess subcutaneous fat to a procedure of extreme sophistication. Plastic surgeons have modified this procedure with the overall goals of improving the aesthetic results and obtaining the ability to mold and shape a patient’s body while focusing on maximizing safety and minimizing complications. Abdominal etching liposculpture represents the continuing evolution of liposuction, creating muscle definition as well as a toned and athletic look.^1-3^

However, although liposuction is very effective for reshaping fatty deposits, the skin may remain lax and may not shrink properly over time. Many devices have been employed to promote and enhance skin retraction after liposuction, including ultrasound-assisted liposuction, power-assisted liposuction, and laser-assisted liposuction. Regardless of which device is utilized, liposuction may lead to the presence of loose skin after fat removal.^4,5^

The main author has systematized a definitive liposculpture approach without the employment of external energy devices; this approach has been called medium definition liposuction. The term medium definition was selected for the sake of differentiating this approach from high-definition liposculpture procedures, which are associated with more etching and higher intensity given the utilization of external energy devices.

The medium definition liposuction approach is based on total fat removal and continuous compression of key areas of the skin by customized handcrafted pads, producing well-controlled fibrosis between the muscle transition zones and the skin. The guided fibrosis is responsible for muscle definition, replacing the utilization of LASER or VASER in select patients.

Sagging skin after liposuction may occur based on skin quality, the amount of fat removal, and the capacity for the skin to be redraped into the “new” shape, exposing the deeper muscle layer. The more fat that is removed, the larger the imbalance between continent (skin) and content (fat). This lack of proportion may produce distortions in the shape of the umbilicus due to the excess skin remaining in the upper abdomen (Figure 1).^6,7^

**Figure 1.**
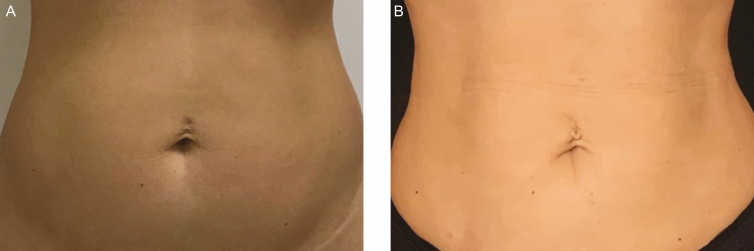
Example of an umbilical deformity (A) preoperatively and (B) 6 months postoperatively demonstrated on this 32-year-old female patient.

Despite having minimal functional importance, the umbilicus is a key aesthetic landmark of the anterior abdominal wall, and it anchors the abdominal skin to the linea alba via the umbilical stalk. The ideal umbilicus is small, nonprotuberant, oval in shape, and relatively high riding. A cosmetically attractive umbilicus should also have a central depression with a superior skin hood, and it should also be vertical in shape.^6,8-10^ The appearance of the umbilicus changes with age and is influenced by the thickness of the abdominal fat as well as by skin quality, weight fluctuations, pregnancies, hernias, and scars.

Sagging of the umbilicus or horizontal deformities are not primary umbilical issues but rather are issues with midline skin laxity cephalad to the umbilicus. To reshape the umbilicus with control and to help with skin redraping in an aesthetically pleasing manner, the primary author has developed a fixation technique of the umbilical tissue associated with suction-assisted liposuction (SAL).

## METHODS

This was a prospective study carried out over 31 months (March 2017 to October 2019) in accordance with the Declaration of Helsinki, and signed written informed consent was obtained.

Sixty-two patients underwent medium definition suction-assisted liposuction with direct needle fixation of the umbilical stalk for the repositioning and prevention of horizontal umbilical deformities, or a “sad-appearing umbilicus.” All patients underwent surgery performed by a single surgeon (G.B.) in a private hospital setting. All patients underwent prospective measurements of the umbilical shape and position before and after the procedure utilizing digital image measurements by Mirror Image software, version 6.0 (Fairfield, NJ). Statistical analysis was performed utilizing IBM SPSS Statistics V26.

Body fat index (BFI), rather than body mass index, is the index of choice for evaluating this population because it defines the ideal candidates for medium definition liposuction. Patients with a low BFI usually exercise regularly, at least 3 times a week. Therefore, these patients have high levels of muscular mass, which can increase the body mass index due to the high lean body mass without necessarily reflecting the percentage of body fat.

The BFI was calculated employing a skinfold caliper in 7 locations: the triceps (the back of the upper arm); pectoral region (the mid-chest); subscapular region (beneath the edge of the shoulder blade); midaxillary regions (midline of the side of the torso); abdomen (next to the belly button); suprailiac region (just above the iliac crest of the hip bone); and quadriceps (middle of the upper thigh). Two measurements were recorded and averaged.

The inclusion criteria of the patients were as follows:

1. Primary absence of skin laxity in the abdomen
2. Hypertrophic and palpable rectus abdominis muscle
3. Body fat index of 20.5% or less.

### Surgical Technique: Medium Definition Liposuction and Umbilical Fixation

The goal of medium definition liposuction is to outline the abdominal muscles in patients by removing the fat in the muscle transition zones (the tendinous intersections of the linea alba, linea semilunaris, and rectus abdominis muscle), revealing and enhancing the deep anatomy and muscular topography, with no need for the employment of any external energy device. The approach is based on the total fat removal and continuous compression of key areas of the skin by customized handcrafted pads placed in strategic areas of the abdomen, producing well-controlled fibrosis between the muscle transition zones and the skin over the course of 30 days.

This guided fibrosis will create a smooth and long-lasting muscle definition, precluding the need to utilize external energy devices for skin retraction. Without employing energy devices for skin tightening, patient selection for medium definition liposuction is limited to fit patients with mild fatty deposits and good skin quality, which is needed for the desired aesthetic end result.

### Skin Markings

**Figure 2.**
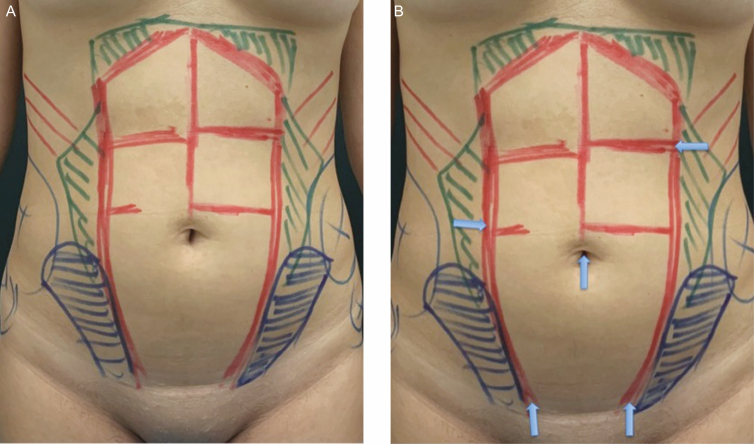
(A) Skin markings and (B) cannula access sites demonstrated on this 29-year-old female patient.

Skin markings were guided by palpation of the muscular tendinous intersections of the rectus abdominis muscle, linea alba, and linea semilunaris. Five access incisions were made in the abdomen utilizing a #11 scalpel, including 1 incision inside the umbilical scar (Figure 2).

SAL/superficial liposuction was performed under regional anesthesia (epidural block) and sedation. The tumescent technique was performed with a 3-mm cannula at a concentration of 1:300,000 lidocaine to epinephrine to achieve adequate vasoconstriction.

A regular perforated liposuction cannula with 3 holes in line (sizes 3, 3.5, and 4 mm) was utilized to perform liposuction in the midline. The surgeon started debulking the deep layers of fat with 4-mm cannulas and then continued toward the mid-lamellar layer and between the muscle groups with 3.5-mm cannulas. Additional superficial liposuction was then performed to define the relevant anatomy for the abdominal muscle groups by utilizing a 3-mm cannula.

To create natural and delicate transition zones between the abdominal muscles, we employed a 3.5-mm cannula to create smooth surfaces and to clean the eventually sharp edges, preventing hard lines. During subdermal liposuction, the nondominant hand was utilized to squeeze and compress the skin to ensure maximal and defined liposuction in order to assist with the creation of the desired indentation required for medium definition liposuction. Last, to induce additional skin tightening at the areas of intersection, subdermal liposuction was performed with the 3-mm cannula with the cannula holes facing up towards the skin.

### Suture Fixation of the Umbilicus

A modified Reverdain’s needle, as proposed by Casagrande et al,^11^ was employed to perform double suturing for umbilical suspension utilizing 3-0 nylon sutures. These cable sutures were bolstered in the umbilicus and in the upper midline.

The intra-umbilical access site, which was utilized during liposuction, was also employed to insert the endoscope (18 cm, 4 mm, 30°C) after liposuction to assess fat removal and guide direct needle fixation. The needle was inserted through the skin at the level of the linea alba in the upper abdomen and was then guided towards the umbilicus where the liposuction access site was previously made. This length was approximately 10 to 15 cm.

**Figure 3.**
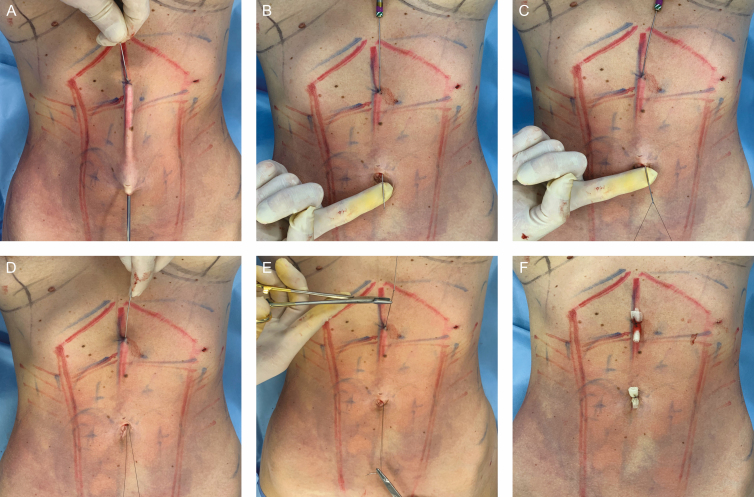
Intraoperative photographs of this 31-year-old female patient. (A) Transcutaneous needle insertion site; (B) needle passing through the access site of the intra-umbilical liposuction incision; (C) 3-0 nylon sutures passing through the hole in the needle tip; (D) needle is pulled back; (E) 2 cable sutures at each end; and (F) the 2 distal cable sutures are tied over a petrolatum gauze bolster.

After the tip of the needle was removed through the umbilicus, a 3-0 nylon suture was passed through the hole in the needle, and then the needle was pulled upwards towards the superior entrance point, exiting out the previous entrance of the needle. Ultimately, there were 2 cable sutures in each end. The 2 ends were then tied over a petrolatum gauze bolster for 2 weeks (Figure 3; Video 1).

### Compression

Compression is one of the main points of medium definition liposuction. After liposuction and umbilical fixation were completed, customized hand-crafted cotton and gauze pads were prepared in the operating room and placed on specific sites of the abdomen to produce specific pressure points of contact between the skin and the underlying fascia. These handcrafted pads were covered with semi-rigid cardboard, which was placed over the anterior abdomen. A traditional liposuction compression garment was placed over the cotton pads and cardboard (Video 2).

At 48 hours, patients removed the original compression pads and received a removable custom compression pad. This second compression pad/second compression garment could be removed so the patient could bathe normally and then was repositioned. We recommended the patient utilize it for at least 1 month after surgery . Utilization of these shaping pads is critical to achieve the best long-term aesthetic results and umbilical fixation.

## RESULTS

Sixty-two patients who underwent medium definition SAL with direct needle fixation of the umbilicus were evaluated through digital photograph measurements by Mirror Image version 6.0 (Fairfield, NJ) preoperatively and 2 weeks and 9 months postoperatively with Statistical analysis IBM SPSS Statistics V26.

All patients were female, and the mean age of the patients was 28.8 years (range, 21-38 years). The follow-up evaluation was performed at 2 weeks and 9 months postoperatively for all patients. The average BFI was 18.7% (range, 16-20.3%). Six patients were postpartum (9.6%).

**Figure 4.**
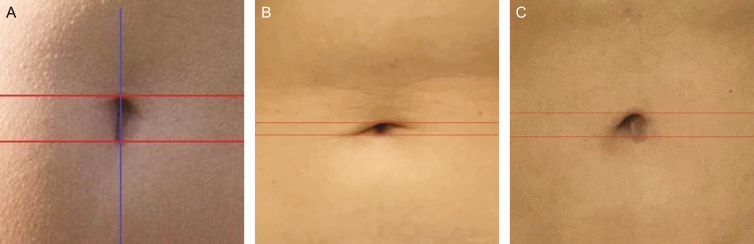
Photographs of this 30-year-old female patient. (A) Umbilical vertical height pattern. (B, C) Change in umbilical vertical height 9 months postoperatively.

The measurement of umbilical height was defined by a longitudinal line crossing the umbilicus in a standing position and 2 horizontal lines that defined the upper and lower edges of the umbilicus. The pre- and postoperative measurements were compared to indicate if any vertical height changes occurred throughout the 9-month follow-up. The authors considered 3 outcomes according to the preoperative vertical height measurements of the umbilicus: (1) unchanged umbilical height; (2) increased umbilical height; and (3) decreased umbilical height. Groups 1 and 2 were considered to have favorable outcomes (Figure 4). Any postoperative decrease in umbilical vertical height was considered umbilicus sagging and represented a poor outcome.

**Figure 5.**
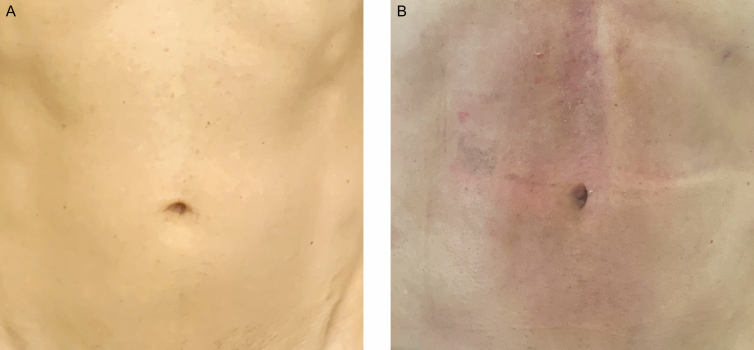
This 27-year-old female patient has a body fat index of 17%. (A) Preoperative and (B) 2 weeks postoperatively, just after fixation with suture removal.

**Figure 6.**
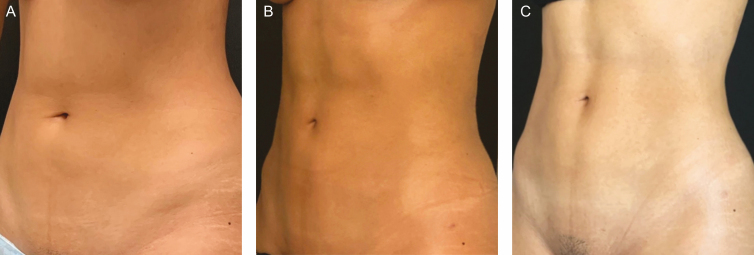
This 29-year-old female patient has a body fat index of 18.5%. (A) Preoperative, (B) 9 months postoperatively, and (C) 12 months postoperatively.

**Figure 7.**
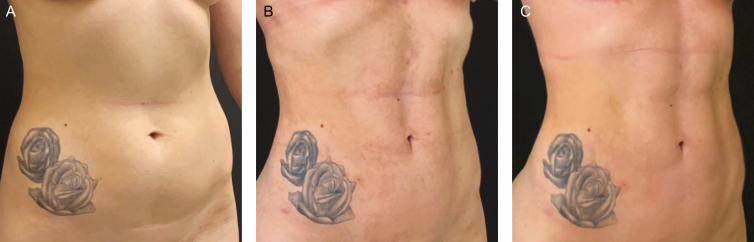
This 31-year-old female patient has a body fat index of 19%. (A) Preoperative, (B) 9 months postoperatively, and (C) 12 months postoperatively.

**Figure 8.**
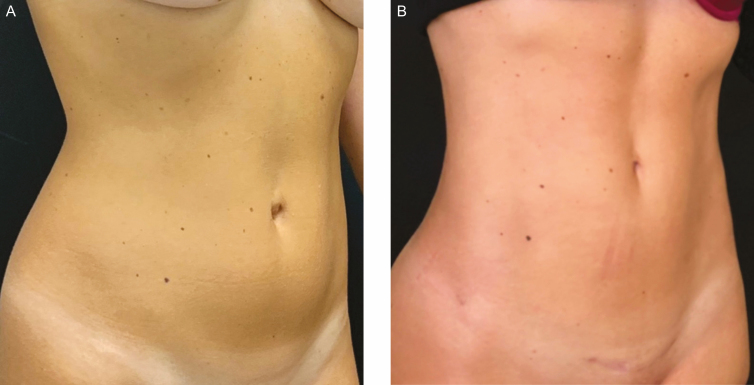
This 32-year-old female patient has a body fat index of 18.8%. (A) Preoperative and (B) 9 months postoperatively.

Two weeks after surgery, the bolstered sutures were removed with complete removal of the suspending nylon sutures, and the umbilical shape was evaluated in terms of the horizontal shape (Figure 5). At the 9-month follow-up, 51 patients had an increase in vertical umbilical height (82.2%), 9 patients displayed maintenance of umbilical height (14.5%), and 2 patients had a decrease in umbilical height (3.2%) (Table 1). There were no major complications, such as necrosis, infection, or seroma. The most frequent complications were minor (n = 17, 27.4%) and are listed in Table 2. Photographs in Figures 6 and 7 show the improvements in umbilical shape at 12-month follow-up, and the 9-month follow-up photograph in Figure 8 shows a vertical umbilical shape.

**Table 1.** Patient Demographics

Case no.	Age, y	Weight, kg	BFI, %	Umbilicus vertical height, mm		
				Preoperatively	2 weeks	9 months
1	28	58	19	21	25.9	21.7
2	29	60	20.3	23	23.7	23.4
3	27	62	21	18.6	21.5	21.2
4	34	59.5	19.5	19.3	21.5	21.2
5	38	59	18	19.8	22.1	21.8
6	30	56.5	17.6	23	23.8	23.6
7	33	62.6	22	22.1	22.9	22.7
8	37	64.5	23	21.2	23.2	23
9	34	60.5	21.3	22.1	18.2	17.9
10	33	61	20.3	19.2	19.9	19.7
11	29	57.5	18	19.3	19.3	19.3
12	23	57.8	19	19.8	20.5	20.2
13	34	56.5	18.5	21.2	21.9	21.7
14	31	57	16	18.1	18.7	18.5
15	38	58.5	20	19.8	22.5	22.2
16	21	60.3	19	19.7	19.7	19.7
17	24	60	18.2	18.9	20.6	20.3
18	32	55.6	17.1	19.3	20	19.8
19	27	58	19	22.1	22.8	22.6
20	26	54.8	17	21.3	21.8	21.7
21	28	55	17.3	19.7	20.4	20.2
22	25	56.6	18.3	21.1	21.1	21.1
23	36	57.3	19.1	20.2	20.9	20.6
24	33	59.2	17.4	19.9	19.9	19.9
25	32	55	16.5	21.1	21.7	21.5
26	25	59.5	19.6	23.2	23.8	23.6
27	32	63.5	21	18.4	21.2	21
28	23	57.5	19.2	18.7	19.5	19.3
29	26	56.8	17.2	19.2	19.9	19.7
30	34	58.5	18.5	20.2	20.4	17
31	24	53	15.3	20.6	21.5	21
32	25	57.4	19.3	18.5	19.3	19.1
33	27	60.1	20.3	19.5	20.4	20.2
34	29	59.5	19.2	21.3	22.4	22
35	31	58.3	19	20.4	21.3	21.1
36	21	57.6	17.5	19.8	20.7	20.5
37	22	56.4	16	21	23.9	23.6
38	32	57.1	16.4	19.3	19.3	19.3
39	23	50	15	18.9	19.7	19.4
40	28	57.3	19	21.2	24.9	24.7
41	29	57	18.1	20.5	21.2	21
42	31	56.4	17.9	20.6	21.4	21.2
43	27	56.9	19	21.4	24.5	24.1
44	24	59.7	20	18.3	18.3	18.3
45	26	55.8	17	19.4	22.7	22.3
46	28	55.7	16.3	22.4	23.3	23.1
47	27	58.3	17.4	21.2	24.2	24.9
48	23	56.3	17.8	22.3	22.3	22.3
49	33	57.4	18	20.1	22.9	22.7
50	26	58.7	19	19.3	20.5	20.2
51	31	57.6	17.9	18.9	20.7	20.4
52	29	59	19	19	22.8	22.5
53	28	56.2	17.5	20.3	21.4	21
54	29	56.5	17.8	21.4	22.6	22.3
55	25	57.3	18	20.2	21.2	20.8
56	30	58.3	19.3	18.6	18.6	18.6
57	28	55.3	16.8	19.8	20.7	20.5
58	34	57.5	19	20.2	21.3	21
59	27	57	18	17.3	20.4	20.1
60	28	58.2	17.8	18.2	18.2	18.2
61	33	57.7	18.5	17.8	18.8	18.6
62	25	57.2	18	18.3	19.5	19.3
Average (range)	28.3 (21-38)	57.4 (50-64.5)	18.7 (16-20.3)	21.4 (17.3-23.2)	25.9 (18.2-25.9)	24.4 (17-24.9)

BFI, body fat index.

**Table 2.** Complications

Complications encountered, n (%)	17 (27.4)
Midline cutaneous dyschromia n (%)	9 (14.5)
Midline skin irregularities n (%)	5 (8)
Umbilicus sagging n (%)	2 (3.2)
Skin ulceration n (%)	1 (1.6)

## DISCUSSION

In 2006, Illouz classified body sites based on the risk of poor cosmetic outcomes after liposuction. The supra umbilical abdominal region was classified as an unforgiving site; even small mistakes during the procedure could cause unfavorable outcomes. The abdominal skin of the upper abdomen immediately above the umbilicus frequently shows excess skin in the upper abdomen, making the umbilicus look “sad.” The skin can roll over the umbilicus, creating a horizontally shaped or sagging umbilicus.^7,12^

Traditionally, the methods employed to prevent sagging of the umbilicus have been patient selection (no loose skin prior to liposuction), small amounts of fat removal from the upper abdomen, the utilization of noninvasive skin-tightening devices, and direct skin removal.^13^ Medium definition liposuction associated with umbilical fixation and the utilization of appropriate postoperative dressing and compression was the method we chose to prevent sagging of the umbilicus. Proper patient selection is key for consistent results of medium definition abdominal etching liposuction. Patients with a regular exercise routine, tight abdominal skin, muscular hypertrophy, and a low BFI are ideal candidates for medium definition liposuction. Therefore, even postpartum and older patients may be candidates if they fit the inclusion criteria . The more fat that is removed, the larger the imbalance between continent (skin) and content (fat). The challenge is to obtain the optimal proportion between fat removal and skin tightening. The dorsum and lateral waist, for example, seem to have better skin recoil than the anterior abdominal skin.

The surgical utilization of ultrasound or LASER has been advocated for boosting skin tightening during liposuction, facilitating the creation of variable degrees of muscle definition. Both technologies have shown a great capacity for eliminating fat and are a good indication for secondary cases, facilitating fat removal and potentially decreasing bleeding and causing less swelling and bruising.

On the other hand, it is known that the amount of skin tightening is unpredictable and depends mainly on the skin quality. The results might vary according to individual features, and attention to the prevention of umbilical sagging has never been demonstrated. Considering the disadvantages of employing external energy devices compared with the utilization of SAL, we found higher rates of thermal injury, fibrosis, and seroma as well as a prolonged procedural time compared with regular SAL.^1,4,14,15^

Abdominal etching, a procedure first introduced by Mentz in the early 1990s, has rarely been reported in the literature.^2^ Currently, “high-definition liposuction” has become synonymous with any type of etching during liposuction regardless of the utilization of external energy devices and definition grading. However, VASER-assisted high-definition liposculpture is a specific technique that was created by Hoyos.^4^ Not all high-definition liposuction procedures follow the original technique, nor do they create a high level of definition.

The technique presented in this paper was named “medium definition liposuction” by the authors. This technique differs from VASER-assisted high-definition liposculpture, because it is based on total fat removal with SAL only and on continuous postoperative compression by customized handcrafted pads, producing well-controlled fibrosis between the transitions in muscle and the skin. This guided fibrosis will create muscle definition, replacing the role of external energy devices in skin retraction. The authors observed that this type of approach produced predictable and reproducible results in the selected population.

The refinement of the umbilical position evolved with the medium definition liposuction procedure. The challenge was to develop a fixation system during the initial healing time (2 weeks) to create enough fibrosis before suture removal. There were several early attempts employing tapes, bolsters, and pads with limited success, probably due to the lack of proper tissue immobilization. The solution was the utilization of suspension sutures, where the anchoring area was fixated during the initial healing and fibrosis process (2 weeks).

The bolstered sutures do not work as a real fixation themselves but rather by immobilizing the tissue immediately after surgery. During this period, the customized pads are compressed, and fibrosis is realigned in the midline. Different attempts to suspend the umbilicus with the utilization of absorbable and internal sutures were performed by the authors without success, causing retractions, distortions, and tissue suspension failures. Therefore, nylon sutures were employed with success for 2 weeks, allowing sufficient time for the tissue to adhere to the skin in the correct aesthetic and anatomic position.

We also noticed that the outcome at 2 weeks predicted success in terms of the umbilical position at 9 months in 96.7% of the patients. A limitation of this technique occurs when aggressive superficial SAL removes the subcutaneous connections between the skin and muscle fascia in the adherence zones, and it can create severe deformities if the transition zones do not heal appropriately. Direct needle immobilization works well when combined with medium definition liposuction and special dressing placement because the procedure creates controlled fibrosis and adhesions in anatomical areas.

Although the main idea for umbilical fixation was initially pursued to prevent sagging of the umbilicus after liposuction, we also observed an improvement in the umbilical shape in most patients, which seems promising. However, the main focus of this umbilical fixation technique is umbilical shape preservation and the prevention of horizontalization. Further studies should be conducted to confirm this approach as a treatment for umbilical sagging.

The limitation of this study is the absence of a control group to establish a causal relationship between navel immobilization and umbilical sagging. However, during the 4 years the main author started performing medium definition liposuction, the author observed that, before 2017 (when we still did not utilize umbilical fixation), the occurrence of umbilical sagging was highly frequent (approximately 60% of the patients had umbilical shape horizontalization at the 6-month follow-up). Over time, with subsequent patients, the main author began systematic immobilization by direct needle fixation, resulting in a decrease in the rate of occurrence of umbilical sagging and distortion.

## CONCLUSIONS

Horizontal deformities of the umbilicus after abdominal liposuction can be prevented by employing needle fixation of the umbilical stalk. The approach has been successful in a selected population of athletic patients. Medium definition liposuction and umbilical fixation have been shown to be effective, safe, and reproducible for the prevention of umbilical sagging.
